# Electrophysiological Responses to Affective Stimuli in Neglectful Mothers

**DOI:** 10.1371/journal.pone.0087808

**Published:** 2014-01-31

**Authors:** Inmaculada León, María José Rodrigo, Ileana Quiñones, Juan Andrés Hernández, Agustín Lage, Iván Padrón, María Antonieta Bobes

**Affiliations:** 1 Faculty of Psychology, University of La Laguna, La Laguna, Canary Islands, Spain; 2 Basque Center on Cognition, Brain and Language (BCBL), Donostia-San Sebastián, Basque Country, Spain; 3 Cognitive Neuroscience Department, Cuban Neuroscience Center, Havana, Cuba; Vanderbilt University, United States of America

## Abstract

Results illustrating an atypical neural processing in the early and late differentiation of infant faces have been obtained with neglectful mothers. The present study explores whether a different pattern of response is observed when using non-infant affective pictures. We examined the event-related evoked potentials and induced delta, theta and alpha activity in 14 neglectful mothers and 14 control mothers elicited while categorizing positive, negative and neutral pictures from the International Affective Picture System. Self-reports of anhedonia and empathy were also recorded. Early posterior negativity, P200 and late positive potential components were modulated by the emotional content of pictures in both groups. However, the LPP waveform had a more delayed and more attenuated maximum in neglectful mothers than in control mothers. Oscillatory responses indicated lower power increases for neglectful mothers than for control mothers in delta (1–4 Hz), theta (4–8 Hz) and lower alpha (8–10 Hz) bands at frontal sites, and a more consistent increase for neglectful mothers in theta and lower alpha bands at occipital sites, especially for negative pictures. These findings help us to better understand the limits of emotional insensitivity in neglectful mothers.

## Introduction

The efficient processing of affective stimuli (e.g., infant faces) and the appropriate response to these stimuli are crucial for adequate parenting. However, this sensitivity pattern to infant cues is not always a given in human mothers. Neglectful mothers are characterized by an insensitive response to their child's demands and a drastic disregard of their child's needs [1], [2]. In fact, negligence is the most prevalent type of child maltreatment, and is associated with child malnutrition, accidents, injuries, untreated health conditions, and developmental delays [3]. The neurological bases of the altered maternal response are receiving increasing attention with a view to better understanding maternal insensitivity to infant cues. However, most studies to date have been done using fMRI (e.g., [4], [5], [6], [7], [8]) while very few have been done with event-related potentials (ERPs) (e.g., [9], [10]). The study by Rodrigo et al. [4] compared the neural response to infant faces in neglectful and control mothers in an attempt to find some neurological bases of maternal insensitivity. Results showed that both groups exhibited greater P200 and late positive potential (LPP) amplitudes at centro-parietal leads in response to crying versus neutral facial expressions, suggesting that infant crying is selected by the brain for sustained processing [11], [12]. However, neglectful mothers displayed a lack of increased face-specific N170 amplitude at temporal leads in response to crying versus laughing and neutral expressions, as well as an overall attenuated brain response in LPP to all infant faces, indicating atypical neural patterns in both early and late processing of infant expressions.

A next step in the analysis of maternal insensitivity in neglectful mothers is to explore whether the findings in Rodrigo et al. [4] are an infant-specific effect or related more generally to affective stimuli as a whole. Mothers must be emotionally prepared and positively motivated to engage socially with their infants, and these states may depend in part on the adequate processing of affective stimuli in general. To this end, the present study analyzes whether ERPs and oscillatory activity may differ in neglectful and control mothers when use is made of affective stimuli other than infant faces, such as those found in the International Affective Picture System, IAPS [13]. These electrophysiological measures allow us to examine the time course of the processing of affective stimuli in the mother's brain, which may have implications for her ability to respond appropriately to them [14]. IAPS pictures and faces differ in terms of their novelty (more unique and novel versus relatively unchanging), complexity (effortful versus automatic response) and sociality (more variable versus inherently social) [15]. Therefore, it is likely that other patterns of neural processing of non-infant affective stimuli may emerge, helping us to reveal further differences between neglectful and control mothers.

There is indirect evidence that could be compatible with the existence of an atypical processing of affective stimuli in neglectful mothers. When a mother is engaged in less than optimal mothering, it is likely that the regulation of the reward and affect system might be affected [16]. In fact, neglectful mothers appear to be less skillful in dealing with emotional communication, because they tend to show lower levels of emotional expression, less emotional perspective taking, and less understanding of their children's emotional displays; they also provide little exchange of emotional information as compared to control mothers [2], [17], [18], [19]. Neglectful mothers also demonstrate a tendency to express no positive affect [20] and to withdraw socially and avoid interpersonal relationships [21].

The electrophysiological correlates of the processing of IAPS pictures in healthy adults have been explored mainly by measuring the three ERP components used in this study: EPN (early posterior negativity), P200 and LPP (late positive potential) (see for reviews [22], [23]). The EPN consists of a negative amplitude deflection between 150 and 350 ms [22], [24]. It has been proposed that EPN indexes “natural selective attention,” such that the evaluation of image features is guided by perceptual qualities that select affectively arousing stimuli for further processing [24]. The P200 is an attention-related component peaking at about 200 ms that is thought to reflect input processing-related attention toward affective pictures that are assumed to be of intrinsic relevance [25], [26]. The later segment of the affective ERP (LPP) is dominated by the P300 component and subsequent positive slow wave lasting for several hundred milliseconds [24]. Whereas the P300 component appears to be transient, the LPP has both a longer duration and a different topography, with activity shifting from parietal electrode sites to more fronto-central sites. P300 and LPP are functionally related as they are typically enhanced after the presentation of motivationally relevant stimuli [11]. However, it is hypothesized that the LPP amplitudes index further allocation of attention resources [11], initial semantic categorization [27], and the representation of stimuli in working memory [28].

The main ERP results obtained with IAPS pictures showed the preferential processing of affective (positive and negative) stimuli as compared to neutral stimuli in the EPN component [24], [29], and in the LPP component across several task requirements [27], [30], [31], [32]. Other studies have shown the preferential processing of pleasant pictures, the so-called “positive offset” bias, in the EPN component [33], [34], in the P200 component [25], [26] and in the P300 component functionally related to the LPP [35], [36]. The “negativity bias” favoring unpleasant stimuli over pleasant and neutral stimuli has also been reported in P200 [32], [37]. However, valence effects are often difficult to disambiguate from arousal effects, since negative stimuli are usually rated as more arousing than positive ones.

The electrophysiological correlates of the processing of IAPS pictures have been less extensively explored by measuring the oscillatory activity, notably the delta, theta and alpha rhythms [38]) that were used in this study. Unlike event-related potentials that always occur in a fixed latency with respect to the stimuli, oscillatory activity, though correlated with the stimuli, may occur at different latencies. Thus, EEG oscillations may provide different functional information related to stimulus processing. The larger EEG amplitude (or power increase) of oscillatory activity for a given sensor is indicative of synchronization in the firing pattern of local groups of neurons [39]. In turn, the power decrease of oscillatory activity for a given sensor is indicative of desynchronization in the firing pattern of the corresponding group of neurons. Although the precise functional meaning of the different frequency bands remains largely undetermined, delta oscillations (1–4 Hz) have been associated with approach-related behavior and reward sensitivity [40]. Theta oscillations (4–8 Hz) have been connected to emotional regulation [40], anxiety, punishment learning [41] and memory [42]. It has been proposed in studies with normal populations that delta and theta oscillations originate in the subcortical motivational circuits [40], while the generation of alpha oscillations (8–12 Hz) is suggested to be more closely related to cortical structures responsible for higher order mental functions, including cognitive regulation [40] and memory [42]. However, the origins of delta and theta oscillations in clinical populations are less certain, given that studies examining oscillatory responses in pathology are still rare. Studies in clinical populations found a diminished delta and theta response in memory tasks in schizophrenics, and a diminished delta oscillatory response in oddball tasks in Alzheimer patients and alcoholics [43].

Evidence from oscillatory activity related to IAPS pictures based on studies with normal populations have shown that delta and theta power are especially pronounced in response to affective stimuli as opposed to neutral stimuli [44], [45], [46], [47], [48]. In addition, increased theta power was also reported in response to negative emotions as compared with positive emotions [46]. Alpha power increases to emotional stimuli were found at frontal sites [38], [45]. Moreover, a frontal-posterior network was reported in which theta and alpha power increases at frontal sites were related to theta and alpha power decreases at occipital sites in response to non-emotional stimuli in memory tasks [42]. The observed pattern of frontal-posterior theta and alpha coherence suggests a regulatory top-down mechanism that functionally connects prefrontal regions with posterior association cortices [42].

Concerning individual differences in oscillatory responses to emotional pictures, the case of alexithymic persons [49] is of special relevance to our study. These persons show several emotional impairments, such as difficulty identifying and describing subjective feelings and difficulty distinguishing between feelings and bodily sensations of emotional arousal. In addition, alexithymia is associated with anhedonia [50], a feature typical of neglectful mothers [9]. Studies comparing oscillatory responses in alexithymic persons with those in control participants showed power decreases at frontal sites in the upper theta band when viewing affective pictures from the IAPS [51].

As individual differences in personality variables such as empathy and anhedonia may influence sensitivity to affective stimuli, it may be helpful to examine how they relate to brain activation patterns. It is likely that decreases in empathy would be at the core of the disregard of the child's needs shown by neglectful mothers [52], [53]; ERP evidence of this is not available, however. Anhedonia, which is the loss of the capacity to subjectively experience pleasure, might lie at the heart of neglectful mothers' tendency to avoid interpersonal relationships and to be unmotivated to pay attention to others [2], [18], [19]. In fact, ERP studies suggest a parallelism between neglectful and depressive mothers in their brain reactivity to emotional stimuli, as a low hedonic tone is characteristic of both populations [9], [10]. However, maternal depression symptoms correlate positively with increased N170 amplitude to infant faces ([10], whereas neglectful mothers exhibit decreased N170 amplitudes in response to infant faces [9]. Neglectful mothers displayed an overall attenuated brain response in LPP to infant faces, which was related to their higher scores in social anhedonia but not in empathy [9].

Based on previous ERP studies, in the present study we predicted that in control mothers, the positive and negative pictures would increase the amplitudes of the EPN and LPP components as compared to the neutral pictures. The P200 response was expected to be modulated by the valence of the stimuli, either positive or negative, in control mothers. By contrast, neglectful mothers would exhibit a lesser emotional modulation of the three potentials. Moreover, based on previous results, we predicted that maternal self-reports of empathy, and especially of anhedonia, would be related to attenuation of LPP.

With regard to oscillatory activity, based on previous studies, we predicted that increases in delta and theta power would be especially pronounced in response to affective stimuli in control mothers. Neglectful mothers' brain responses could follow the atypical pattern of alexithymic persons, showing decreases in the upper theta band at frontal sites. In control mothers, we would also expect to see theta and alpha power increases at frontal sites in response to emotional stimuli and decreases at occipital sites according to the frontal-posterior network found in response to non-emotional stimuli in memory tasks. A more atypical pattern in the frontal-posterior network could be expected for neglectful mothers.

## Methods and Instruments

### 2.1. Participants

A total of 30 white mothers (two were later discarded because of technical problems) participated in the data collection and gave written consent according to the protocol approved by the Ethical Committee of the University of La Laguna. They were all attending a preventive parenting program delivered by the municipal social services, as they came from poor socioeconomic backgrounds [54]. The same groups of mothers (14 neglectful and 14 control) had participated in the first ERP study using infant faces as affective stimuli [9], with a two-week interval between the two studies. Neglectful mothers were drawn from a pool of 36 at-risk mothers (including 27 neglectful mothers), and control mothers were drawn from a pool of 25 mothers. The group of neglectful mothers was highly homogeneous: a) their primary identified problem was substantiated neglect of a child under five years old according to the Maltreatment Classification System [1]; b) all mothers in this group exhibited the three main subtypes of neglect: physical neglect, lack of supervision and educational neglect; c) all had a history of maltreatment, while none had severe mental health problems (e.g. depression), were drug abusers and/or had a low IQ according to the social services. If they had had such problems, the social services would have received a report from the mental health services. All mothers in the control group had at least one child under five years old, a confirmed absence of neglectful behavior in the social services records and no severe mental health concerns. An analysis of key demographic variables revealed that the mothers in the neglectful and the control groups did not significantly differ on any variable (Table 1).

**Table 1:** Sociodemographic characteristics of mothers and personality variables in neglectful and control groups.

	Neglectful group (N = 14)	M (SD) or %	F/χ2/t
	M (SD) or %	Control group (N = 14)	
Mean age of mother	33.6 (7.2)	36.6 (6.4)	1.33
Number of children	2.8 (1.2)	2.2 (0.9)	2.83
Mean age of the target child	3.5 (1.8)	3.0 (1.6)	0.47
Rural areas (%)	47.1	42.9	0.57
Two-parent family (%)	82.4	100	2.54
Level of education (%):			
Primary	58.8	50.0	1.52
Secondary school	41.2	41.7	
>Secondary school	0.0	8.3	
Unemployed (%)	76.9	50.0	1.55
Anhedonia			
Physical	17.54 (5.42)	11.80 (6.46)	2.521*
Social	14.15 (7.72)	7.36 (4.08)	2.888**
Interpersonal reactivity index			
Perspective-taking	23.21 (5.17)	25.47 (4.06)	−1.307
Fantasy	18.50 (3.67)	21.93 (5.98)	−1.845
Empathic concern	27.28 (4.17)	30.66 (3.57)	−2.345*
Personal distress	18.93 (4.71)	16.67 (2.66)	1.605

*Note*: p<.05*; p<.001**.

### 2.2. Stimulus materials and task

Seventy-two pictures, twenty-four for each condition, were selected from the IAPS (see [13]) based on their valence and arousal ratings and avoiding examples of infant faces. The IAPS slide numbers were as follows: positive 4520, 5200, 1463, 4641, 8200, 5831, 7475, 4660, 4653, 1920, 2070, 4606, 4650, 4599, 4652, 4680, 7230, 4250, 8510, 7350, 1710, 4611, 7330, 4608; negative 6230, 3168, 3130, 6370, 6190, 6200, 3150, 1120, 1300, 3100, 3060, 1200, 1120, 6020, 9040, 2120, 3010, 1070, 3000, 1050, 3530, 1090, 1274, 9490; and neutral 2200, 7090, 7100, 7050, 7570, 5510, 5500, 7010, 2215, 5520, 7150, 2383, 7700, 5534, 7950, 2190, 7080, 2515, 5535, 2516, 7130, 7550, 7500, 7000. The final set was presented twice in a random order, with no more than two stimuli of the same stimulus category being presented in succession. Positive pictures included sports, pets, and erotic themes; negative pictures included mutilated bodies and adults in risky scenes; and neutral pictures were of common objects or adults in everyday scenes. The normative valence and arousal ratings of the selected IAPS pictures for women were the following: for positive stimuli (24 items): valence = 7.3 (1.5), arousal = 5.2 (2.3); for negative stimuli (24 items): valence = 2.5 (1.5), arousal = 6.8 (2.0); for neutral stimuli (24 items): valence = 5.2 (1.4), arousal = 3.7 (2.0). Stimulus selection was based on previous studies establishing that differences in affective content reliably discriminate in terms of their evaluative, somatic, autonomic and electrophysiological responses [11], [55]. The content groups were selected such that there was almost no overlap in the IAPS normative affective valence ratings, i.e. the three stimulus content groups were distinct and representative of affect type. However, the distributions of arousal ratings for pleasant and unpleasant pictures were not symmetrical, because in the IAPS distribution the low arousal end of the unpleasant pictures range was less extended than for pleasant pictures. Participants' ratings of valence and arousal dimensions in the present study are reported in the results section (Table 2). Images were displayed in the center of the screen for 2000 ms. The inter-trial interval varied between 2000 ms and 3000 ms. Ten pictures, different from the experimental trials, served as practice trials. Mothers were instructed to view each picture and to respond after stimulus offset by categorizing it either as a positive, negative or neutral picture by pressing a three-way response button. After the ERP task, valence and arousal subjective ratings on a 0–9 Likert scale (0 = very unpleasant/no activating at all; 9 = very pleasant/very activating) were obtained using a written version of the Self-Assessment Manikin (SAM; [56]).

**Table 2:** Behavioral and self-rating measures by group and categories of pictures.

	Neglectful mothers			Control mothers		
	Positive M (SD)	Negative M (SD)	Neutral M (SD)	Positive M (SD)	Negative M (SD)	Neutral M (SD)
RT (ms)	2140 (816)	2171 (882)	2231 (794)	2276 (755)	2242 (795)	2379 (782)
Valence	6.8 (1.4)	2.2 (1.1)	4.8 (0.5)	5.8 (1.6)	3.9 (1.8)	5.1 (1.0)
Arousal	4.5 (1.6)	6.8 (1.8)	2.9 (1.1)	4.3 (1.4)	6.2 (1.6)	4.5 (2.0)

### 2.3. Personality measures

**Empathy:** The Interpersonal Reactivity Index (IRI; [52]) is a 28-item self-report questionnaire, which contains two cognitive scales (Perspective-Taking and Fantasy) and two affective scales (Empathic Concern and Personal Distress). Participants respond to each item using a 5-point Likert-type scale ranging from 0 (does not describe me well) to 4 (does describe me well). The Perspective-Taking Scale assesses the tendency to spontaneously adopt the psychological point of view of others (e.g., “I try to understand my friends better by imagining how things look from their perspective”). The Fantasy Scale measures the respondents' tendency to identify with fictional characters, such as characters in books, movies, or plays (e.g., “When I watch a good movie, I can very easily put myself in the place of a leading character”). The Empathic Concern Scale taps the respondents' feelings of warmth, compassion, and concern for others (e.g., “I often have tender, concerned feelings for people less fortunate than me”). The Personal Distress Scale assesses self-oriented feelings of anxiety and discomfort resulting from tense interpersonal settings (e.g., “Being in a tense emotional situation scares me”). Individual scores were calculated for each subscale. The available Spanish version of the scale was used [57], which has shown adequate internal consistency (from 0.67 to 0.80).

**Anhedonia:** Anhedonia was assessed using the revised versions of the Physical Anhedonia Scale (RPAS, [58]) and the Social Anhedonia Scale (RSAS, [59]), with true-false self-report measures, which provide indices of the pleasure derived from physical and social-interpersonal sources, respectively. The Revised Physical Anhedonia Scale (61 items) assesses a self-reported deficit in the ability to experience pleasure from typically pleasurable physical stimuli such as food, sex, and settings (e.g., “Beautiful scenery has been a great delight to me,” keyed false). The Revised Social Anhedonia Scale (40 items) assesses deficits in the ability to experience pleasure from non-physical stimuli such as other people, talking and exchanging expressions of feelings (e.g., “Having close friends is not as important as many people say,” keyed true). Both scales have shown considerable reliability (from 0.77 to 0.88) in control and neglectful Spanish mothers [9].

### 2.4. Apparatus and recordings

The EEG was recorded on a MEDICID 5 system (Neuronic SA, Havana) from nineteen channels according to the 10–20 system using disk electrodes contained in a cap (Electro-cap Systems). The EEG was measured with respect to electrodes placed on the mastoids, but an average-reference transformation was used off-line for the analysis of the EPN component. Using the mastoids as reference for EPN would have cancelled it, because of their proximity to the occipito-temporal electrodes where this component is measured [60]. The average reference permits a more accurate estimation of the possible amplitude effect in this component, even with a low-density array like the one used in our study. The impedance of all electrodes was kept below 10 kΩ. Additional electrodes were used to record horizontal and vertical eye movements (electrooculogram, EOG). The signals were amplified by a factor of 1000 and filtered between 0.05–30 Hz. The EEG was continuously recorded (sampling rate 200 Hz).

### 2.5. ERP analyses

The ERP recording was time-locked to the stimulus onset. Epochs were created beginning 100 ms prior to stimulus onset and continuing for 1000 ms. The following 1000 ms before the stimulus offset allowed for the preparation of the behavioral response that should be postponed after the stimulus offset. All epochs showing peaks with abnormal voltage values (above 50 µv) were rejected. Thereafter, all EEG segments were visually inspected and those contaminated by any other artifact were manually eliminated off-line. Means and standard deviations for number of trials did not differ by group: Positive: 32.4 (11.1), Negative: 31.4 (10.9) and Neutral: 33.8 (11.1) for the Neglectful group; Positive: 35.4 (9.4), Negative: 36.1 (8.5) and Neutral: 36 (8.9) for the Control group.

The choice of time windows for the ERP analyses was based on theoretical and statistical criteria. First, we identified the three ERP components described in the literature: EPN, P200 and LPP. Second, a statistical criterion was applied to identify significant points and to correct for multiple comparisons. Specifically, the non-parametric statistical method of permutations (included in the Neuronic EP Workstation package) was used to estimate pairwise t-test comparisons between levels of a variable (the three categories of picture) at each data-point. Third, only segments reaching the statistical criterion in the permutation analyses and with a temporal overlapping of the significant points for the two comparisons (positive vs neutral and negative vs neutral) were selected for further ANOVA analyses, which also included the group of mothers as a variable. Greenhouse-Geisser correction was used where appropriate (only when the Mauchly Test for sphericity was significant).

**EPN:** A negative shift was observed in the occipito-temporal region within the time window from 205–330 ms. The mean amplitude scores in the EPN window were analyzed by means of 2×2 repeated-measure analyses of variance (ANOVA), with Group (control and neglectful mothers) as the between-subject factor and Category of picture (positive, negative and neutral) as the within-subject factor. Four temporo-occipital electrodes (T5, T6, O1, O2) were used for the analyses. Data for the selected electrodes in the ANOVAs were averaged. A similar analysis was performed for the latency scores, using the time-point of maximal amplitude in the defined area as the dependent measure.

**P200:** An attention-related component showing augmented amplitudes over bilateral fronto-centro-parietal sensors was observed in a time window from 200–300 ms. Mean amplitude in the window selected was used as a dependent variable. Data were examined via 2×2×3 ANOVA, with Group (control and neglectful mothers) as the between-subject factor, and Category of picture (positive, negative and neutral picture) and Region as the within-subject factors. Three areas of interest, with three electrodes each, were included as a fixed within-group factor (Region). The three areas were: Frontal (F3-Fz-F4), Central (C3-Cz-C4), and Parietal (P3-Pz-P4) electrodes. Data for the selected electrodes in the ANOVAs were averaged. A similar analysis was performed for the latency scores, using the time-point of maximal amplitude in the defined area as the dependent measure.

**LPP:** A third potential showed an area of augmented amplitudes over bilateral centro-parietal sensors around 550 ms that lasted for several hundred milliseconds. Frontal sensors showed augmented amplitudes as well, so they were also included in the analyses. Likewise, remarkable differences were observed between latencies of the LPP waveforms by group, with peaks observed at a later latency in the neglectful group than in the control group. The mean amplitude scores for the LPP area (between 600 to 780 ms) were analyzed using ANOVA with the same factors and design (2×2×3) as for P200. Data for the selected electrodes in the ANOVAs were averaged. A similar analysis was performed for the latency scores, using the time-point of maximal amplitude in the defined area as the dependent measure.

### 2.6. Time-frequency analyses

Following Hald et al, [61], the time-frequency representation (TFR) was computed for every trial by convolving a Morlet wavelet with a signal with a 7-cycle width, with frequencies ranging from 1 to 30 Hz, in 0.5 Hz steps. To isolate the induced-type oscillations (non-phase locked) from the effects of the (evoked-type) ERP components, we computed the average ERP for every subject and subtracted this average from each single trial. Averaged TFRs were then computed by averaging the power over the single trials for each condition and subject separately. The resulting average power values were then expressed as the percentage of power increase or decrease with respect to the power computed in a 100 ms prestimulus baseline interval [39].

At this step, the time-frequency data comprised power change maps, relative to baseline, for each condition and subject. Next, the statistical comparison of conditions was performed. Here we tested the conjunction hypothesis using a procedure previously desribed by Onton et al. [62] for time-frequency analysis. The conjunction hypothesis posits consistency of activations in the power maps across subjects, and has the advantage of controlling for variability in map scale across subjects [63], [64]. First, relative power change maps were converted to binary maps (0 non-active, 1 active) for each subject and condition using a mixture density model that classifies the generators into a null (non- active) and an alternative (active) distribution using the local False Discovery Rate (FDR) algorithm [65] at a *p* level of 0.05. Next, the conjunction statistic was computed as the proportion of subjects with an active point at a particular time-frequency. For all time-frequency points, group level *p*-values were obtained using binomial density [63] with *p* = 0.05, and significant time-frequency points were extracted at an alpha level of 0.05. To avoid false positives in a situation involving multiple comparisons, only those clusters with at least 15 activated and adjacent Time-Frequency points were considered as significant.

### 2.7. Behavioral and self-rating measures

We recorded reaction times and accuracy in the classification task of pictures according to their emotional content. SAM ratings (arousal and valence dimensions) were also recorded (using a 0–9 scale). Both sets of data were submitted to ANOVAs, with Group (control and neglectful mothers) as a between-subject factor and Category of picture (positive, negative and neutral) as a within-group factor.

### 2.8. Analysis with personality measures

Personality differences between neglectful and control mothers were explored by means of *t* tests. Personality scores in empathy and anhedonia were included as covariates in the repeated-measures analyses (ANCOVAs) to examine whether group differences in the mean levels of the personality variables might explain variability in the amplitudes of the three ERP components. We then performed correlations between those personality measures and the mean ERP amplitudes, controlling for multiple correlation bias. The same sequence of analyses was followed with self-report personality scores and power scores in delta, theta and alpha oscillation bands.

## Results

### 3.1. Behavioral and self-rating analyses

Participants correctly classified an average of 80% of the stimuli and there were no significant differences by group or emotional content. Results for mean reaction times were not significant by Group of mothers, only for Category of picture, *F*(2, 52) = 5.92; *p*<.05, ε = 0.61, *partial η^2^* = 0.10. In both groups, shorter reaction times were obtained for classifying positive and negative pictures as compared with neutral pictures (positive<neutral: *t*(27) = −2.6; p<.05; negative<neutral: *t*(27) = −2.5; p<.05). Results for subjective ratings on valence and arousal were not significant by Group of mothers, only for Category of picture (valence, *F*(2,52) = 27.48; p<.001, *partial η^2^* = 0.22; arousal, *F*(2,52) = 17.66; p<.001, *partial η^2^* = 0.17). In both groups a linear decrease in self-rated valence was observed for positive, neutral and negative pictures. Finally, participants rated the neutral pictures as less arousing than the positive and negative ones. All comparison for valence and arousal were significant at p<.05 (see Table 2).

### 3.2. ERP effects

**Figure 1:**
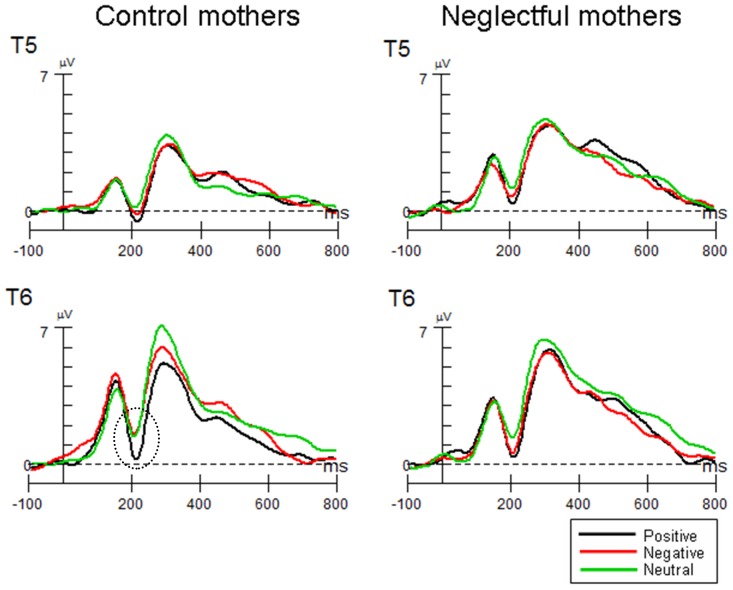
EPN component in control and neglectful mothers to positive, negative and neutral pictures. Electrodes at T5, T6, O1 and O2 were selected for representing the component. Positive and negative pictures elicited significantly larger negativity than neutral pictures in both groups of mothers.

**Early Posterior Negativity (EPN):** Affective pictures elicited significantly larger negativity than neutral pictures in both groups of mothers over temporo-occipital regions, *F*(2, 52) = 6.07; *p*<.01, *partial η^2^* = 0.09 (Figure 1). *Post hoc* tests revealed that the positive and negative pictures were associated with an increased negativity relative to neutral pictures (positive<neutral: *t*(27) = −3.69; *p*<.01; negative<neutral: *t*(27) = −2.60; *p*<.05), whereas positive pictures did not significantly differ from negative pictures. The Group x Category interaction was not significant (*F*(2, 52) = 0.08; *p*>.05).

As for the latency, no significant differences were found between neglectful and control mothers, *F*(1, 26) = 0.18; *p*>.05. Differences in latency were not found by category of pictures, *F*(2, 52) = 1.95; *p*>.05. Likewise, the Group x Category interaction was not significant (*F*(2, 52) = 0.70; *p*>.05).

**Figure 2:**
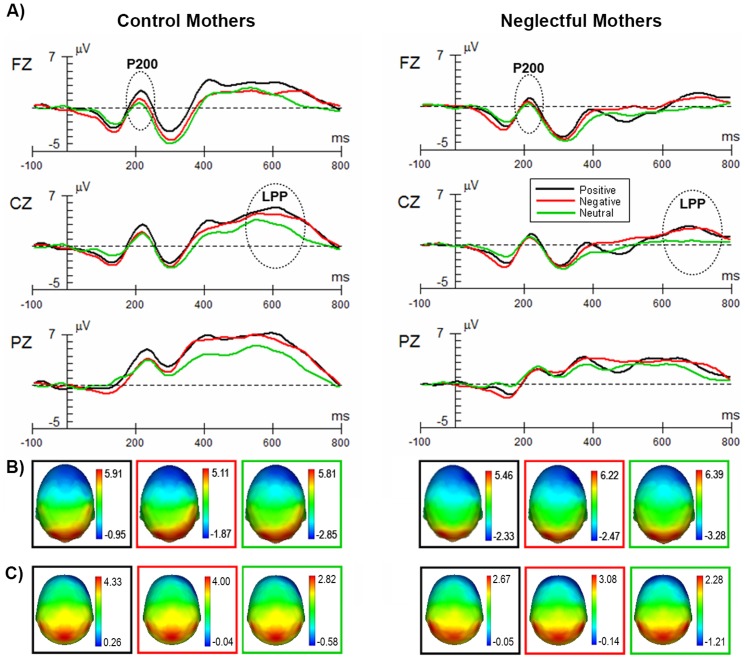
P200 and LPP components in control and neglectful mothers to positive, negative and neutral pictures. (A) Grand-averaged ERP waveforms corresponding to the midline electrodes Fz, Cz and Pz. The P200 was more pronounced for positive pictures than for neutral pictures at the frontal area in both groups. LPP was more pronounced for positive and negative pictures than for neutral pictures at the fronto-centro-parietal areas in both groups. Dotted ellipses indicate the maximum amplitude for each group. Scalp topographies are given (B) for P200 and (C) for LPP, showing different amplitudes by category of pictures (black, red and green squares), and by group (control-neglectful).

**P200 component:** This component was modulated by the interaction between Category of picture and Region, *F*(4, 104) = 3.07; *p*<.05, ε = 0.57, *partial η^2^* = 0.11. Reliable differences between categories of pictures were only found in the frontal region for both groups (Figure 2B). In this region, positive pictures prompted a higher positivity with respect to neutral ones, *t*(27) = 2.58; *p*<.05, whereas the other comparisons were not significant (Figure 2A). The amplitude of the potential changed by Region in both groups, *F*(1, 52) = 27.69; *p*<.001, ε = 0.57, *partial η^2^* = 0.49. The largest positivity was observed at the parietal region, in relation to the central region, with the lowest positivity observed at the frontal region (at *p*<.001). The Group x Category interaction was not significant (*F*(2, 52) = 0.97; *p*>.05).

As for the latency, no significant differences were found between neglectful and control mothers, *F*(1, 26) = 1.13; *p*>.05. Differences in latency were found by Region, *F*(2, 52) = 29.33; *p*<.001, ε = 0.76, *partial η^2^* = 0.44. Significant differences were found between the central>frontal>parietal regions at *p*<. 001. The Group x Category interaction was not significant (*F*(2, 52) = 0.85; *p*>.05).

**Late Positive Potential (LPP):** LPP was modulated by the Category of picture for both groups, *F*(2, 52) = 16.54; *p*<.001, *partial η^2^* = 0.14, (Figure 2A). Both positive and negative pictures prompted a greater positivity than neutral pictures in both groups (positive>neutral: *t*(27) = 4.94; p<.001; negative>neutral: *t*(27) = 5.35; *p*<.001). However, there was a Group effect, as the waveform for the neglectful mothers appeared attenuated in amplitude across the three categories of pictures with respect to that of the control mothers, *F*(1, 26) = 4.34; *p*<.05, *partial η^2^* = 0.88. The ERPs associated with all pictures showed an enhanced positivity at fronto-centro-parietal sensors (see Figure 2C). The amplitude of the potential changed by Region in both groups, *F*(2, 52) = 62.85; *p*<.001, ε = 0.67, *partial η^2^* = 0.23. The largest positivity was observed at the parietal region, in relation to the central region, with the lowest positivity observed at the frontal region, at *p*<.001. The Group x Category interaction was not significant (*F*(2, 52) = 0.48; *p*>.05).

As for the latency, a more delayed maximum was found for neglectful mothers than for control mothers, *F*(1, 26) = 7.62; *p*<.05. Differences in latency were also found by Region, *F*(2, 52) = 16.73; *p*<.001, ε = 0.71, *partial η^2^* = 0.16. Significant differences were found between the central>frontal>parietal regions at p<. 001. The Group x Category interaction was not significant (*F*(2, 52) = 0.42; *p*>.05).

### 3.3. Relations of personality scores with ERP and TF data

The neglectful mothers scored significantly higher on Physical and Social Anhedonia and lower on Empathic Concern than the control mothers (Table 1). As for the relations of personality with ERP and TF data, the inclusion of self-ratings as covariates did not show any significant group differences or any significant within-subject effects in ERP amplitudes and latencies or in power TF measures in delta, theta and alpha bands. No significant corrected correlations were found between personality self-reports and ERPs or the different power values obtained by sensor.

### 3.4. Time-frequency effects

**Figure 3:**
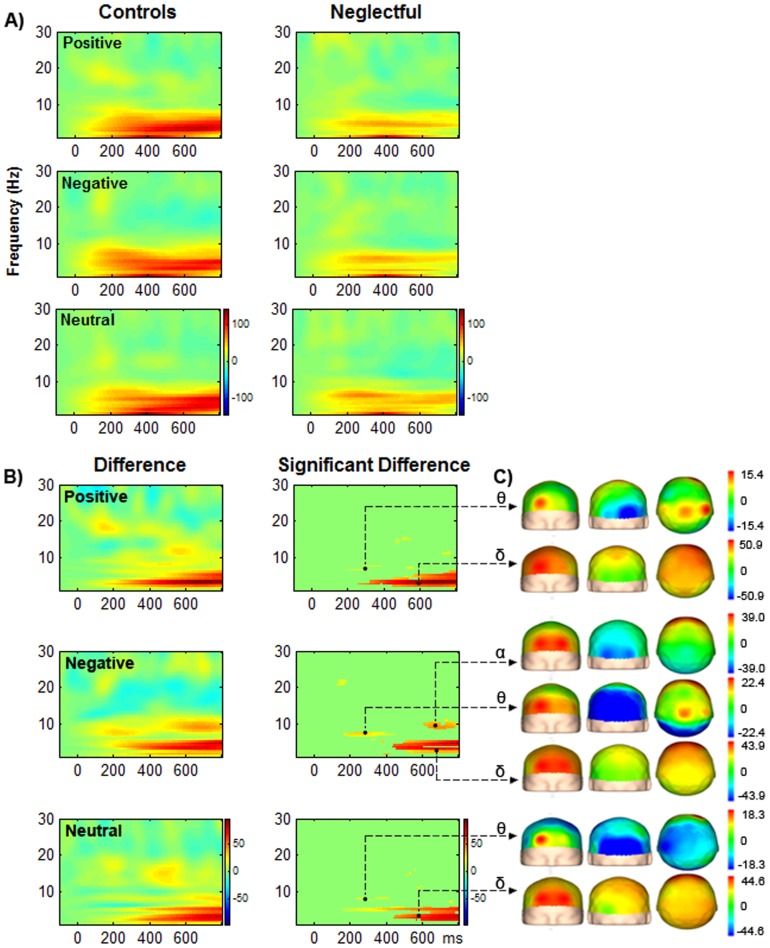
Time-Frequency representations (TFRs) of the power changes in control and neglectful mothers to positive, negative and neutral pictures. A) TF plots are given for C3 depicting power changes in the three categories of pictures for control and neglectful mothers, with red representing power increase and blue depicting power decrease relative to baseline. B) Plots are given for differences between the two groups of mothers for each category (left-hand side) and the statistically thresholded TFRs (p<.05; right-hand side). C) Scalp topographies are given for significant power changes between the two groups. In the delta, theta and lower alpha bands, a higher power increase is observed for control mothers than for neglectful mothers at frontal sites (time windows after stimulus onset from 300–800 ms, 200–400 ms, and 600–800 ms, respectively). A higher power increase in the theta band, especially in negative pictures, and in the lower alpha band for negative pictures only is observed at occipital sites in neglectful mothers as compared to control mothers. The scaling was optimized according to the maximum value. The original values of control versus neglectful group differences in power increases per condition and band were the following: For positive stimuli (delta from 50.9 to 3.5; theta from 15.4 to −20.8); for negative stimuli (delta from 43.9 to 1.2; theta from 22.4 to −58.1; lower alpha from 39.0 to −34.3); for neutral stimuli (delta from 44.6 to 8.3; theta from 18.3 to −36.4).

As illustrated in Figures 3A and B, the results of induced effects showed that the group significantly modulated the power changes within the frequency band of delta and theta for all categories of stimuli and within the lower alpha band for negative stimuli. Power increases in delta band were more consistently present at frontal sites in control mothers than in neglectful mothers for all stimuli (see Figure 3C). The same pattern was obtained for theta band for all stimuli, with the pattern being especially robust for negative pictures. However, the highest power increases in theta band were more consistently present at occipital sites, especially in neglectful mothers. Consequently, the higher the increases at occipital sites, the lower the increases at frontal sites in neglectful mothers and vice versa in control mothers. Similarly, a higher increase in lower alpha band at occipital sites in neglectful mothers was associated with a lower increase at frontal sites. Alpha effects were only significant for negative stimuli. Thus, we observed the frontal-posterior pattern in theta and alpha bands in control mothers and an atypical reversal pattern in neglectful mothers, as expected. Oscillatory effects were more pronounced later in delta and alpha bands overlapping with the LPP interval (from 600 ms), and relatively earlier in theta band overlapping with the P200 interval (around 200 ms).

## Discussion

This research combines the use of electrophysiological and self-report measures to examine the brain patterns in neglectful mothers in response to IAPS pictures as compared to control mothers. Concerning the emotional modulation of ERP at different stages, the morphology, time windows, amplitudes, and regions of augmented amplitudes are comparable to the respective ERP patterns obtained in other studies with IAPS stimuli. The EPN and LPP results supported the natural selective attention to emotional stimuli, according to which positive and negative stimuli are primed by their biological significance for further processing, as compared to neutral stimuli [66], [67], [68]. Thus, in several studies, the preferential processing of affective stimuli was observed in the EPN component [24], [29] and in the LPP component [27], [30], [31], [32], [33]. In turn, the P200 results supported the positive offset hypothesis [11], [25], [34], as increased P200 to positive versus neutral stimuli was found in the frontal sensors. This effect can be interpreted as a genuine valence effect (e.g., positivity bias), as arousal ratings were higher for negative pictures than for positive pictures.

As for group differences, neglectful mothers did not show any atypical neural processing as compared to control mothers in the early differentiation of affective and neutral stimuli, in either EPN or P200 potentials. Group x Category interactions were not significant. Therefore, it is likely that the affective stimuli featured by the IAPS may have a privileged status, as they were selected for differential early processing in both groups of mothers. In addition, the larger LPP amplitudes for emotional pictures than for neutral pictures obtained in both groups of mothers suggest that affective stimuli underwent more elaborate analysis at a later stage of emotional processing [69].

However, neglectful mothers showed both attenuated LPP amplitudes and delayed latencies in comparison to control mothers in response to all categories of IAPS stimuli. In an ERP study performed with mothers viewing infant faces, it was found that higher amplitudes on the late positive P300, functionally related to the LPP, were associated with mothers' perceptions of the parent-child relationship as being positive and influential for their children's psychological development [14]. Consistently, in both this study and a previous one with the same sample [9], we found that attenuated LPP responses to emotional stimuli (IAPS and infant faces) were more prevalent in neglectful mothers, who usually present inadequate patterns of parent-child relationships and a severe disregard of their child's developmental needs [1], [2]. Therefore, inadequate parenting seems to be associated with inefficient late processing of emotional signals.

Individual differences in empathy and anhedonia in neglectful mothers were not related to ERP and TF brain responses, although neglectful mothers did show lower empathic concern and higher anhedonia than control mothers. In the study by Rodrigo et al. [9], in which the same mothers were exposed to infant faces, social anhedonia did contribute to explaining the attenuation observed in the LPP in the neglectful group as compared to the control group. A possible explanation for this is that the social nature of anhedonia more closely matches that of the infant stimuli than the IAPS pictures selected for this study. This suggests that anhedonia effects on LPP in neglectful mothers could be sensitive to the type of emotional stimulus.

With regard to induced activity, group effects consistently appeared in the time-frequency analyses, providing a complementary picture of the brain differences between neglectful and control mothers. Oscillatory responses indicated lower power increases for neglectful mothers than for control mothers in delta, theta and lower alpha bands at frontal sites, and a more consistent increase for neglectful mothers than for control mothers in theta and lower alpha bands at occipital sites. The lower increase in delta may reflect problems in the activity of motivational systems associated with the approach-related action and reward sensitivity in neglectful mothers, which may result in a poor processing of emotional and neutral stimuli at the attentive level [40]. As mothering represents a motivated behavior which has an impact on and interacts with reward and affect systems [16], these results are consistent with the lack of motivated interest and disregard that neglectful mothers show towards their children.

We also found a reverse pattern for theta and alpha bands at different sites: the higher the increases at frontal sites, the lower the increases at occipital sites. More importantly, this pattern was found in control mothers, whereas in neglectful mothers the pattern was the opposite: the higher the increases at occipital sites, the lower the increases at frontal sites. The frontal-posterior network, which reflects a binding process between anterior and posterior regions, was also found in healthy adults in response to non-emotional stimuli [42]. In our case, the theta and lower alpha effects could reflect group differences in the activity of regulatory networks that facilitate a higher engagement in top-down processing of emotional stimuli in controls as compared to neglectful mothers. However, the alpha effect that was mainly observed with negative stimuli should not be interpreted as a genuine negative stimuli effect, as arousal effects might also be involved. A similar disrupted frontal power variation in theta band, for affective stimuli only, was also found in alexithymic persons [51], which is compatible with the disturbed emotional profile shared with neglectful mothers. A functional neuroimaging study has also delineated a frontal-posterior network responding to IAPS stimuli [70]. In this study, negative pictures recruit the lateral parts of the prefrontal cortex, whereas ventromedial prefrontal regions support processing of positive pictures. Negative pictures activate the occipital cortex regardless of arousal level, whereas positive pictures activate this region only when they are highly arousing. This means that the increased engagement of the visual cortex is especially salient for negative pictures. In the light of these results, it would be interesting to further examine our findings on the higher oscillatory activity in occipital sensors in neglectful mothers to confirm their relevance as an indicator of inadequate parenting.

Several limitations of the present study and future directions should be mentioned. Although participants were the same in both studies, infant faces and affective IAPS pictures were not examined simultaneously, but two weeks apart. Thus, comparison between responses to infant faces and affective pictures could only be done indirectly. Future research could address this direct comparison with a bigger sample size. In addition, we do not know whether the oscillatory pattern found here differed from the one that would have been obtained for infant faces, since time-frequency analyses were not performed in the first study. Therefore, the diminished activity found in the delta band and the atypical frontal-posterior network found in the theta and alpha bands in neglectful mothers merit further investigation using infant faces and other social stimuli, such as adult faces, to better disentangle what is specific to inadequate parenting.

## Conclusions

In this study we have taken one step forward by starting to define the boundaries of emotional insensitivity in neglectful mothers. Thus, we have shown that the early emotional modulation in neglectful mothers is sensitive to the differences between faces and IAPS stimuli, suggesting that what seems to be specifically affected is the emotional recognition of infant faces. By contrast, the late processing deficits in neglectful mothers are found regardless of the stimulus type, lending support to the existence of a global atypical pattern in the elaborated processing of both types of stimuli. The atypical oscillatory dynamics in neglectful mothers could reflect an anomalous function in the motivational system and in the top-down regulation of emotional stimuli.

A growing understanding of the neural underpinnings of neglectful behavior would help to identify biomarkers that reliably index maladaptive maternal processes. Animal models have shown that non-attentive care in rats is an environmental epigenetic factor that can negatively affect gene expression in their offspring [71]. However, research has also shown that these negative effects can be reversed with a good maternal care [71]. Results such as these generate a hope that psychological programs, designed to sensitize neglectful mothers to infant signals, can reverse the devastating effects of their lack of affection with their babies. The use of biomarkers would help to test, at a more basic neurological level, the effectiveness of such psychological interventions.
